# Scavenging Properties of Plant-Derived Natural Biomolecule Para-Coumaric Acid in the Prevention of Oxidative Stress-Induced Diseases

**DOI:** 10.3390/antiox10081205

**Published:** 2021-07-28

**Authors:** Shubhadeep Roychoudhury, Barnali Sinha, Birupakshya Paul Choudhury, Niraj Kumar Jha, Partha Palit, Surekha Kundu, Subhash C. Mandal, Adriana Kolesarova, Mokhtar Ibrahim Yousef, Janne Ruokolainen, Petr Slama, Kavindra Kumar Kesari

**Affiliations:** 1 Department of Life Science and Bioinformatics, Assam University, Silchar 788011, India; barnalisinha1997@gmail.com (B.S.); birupakshyapc@gmail.com (B.P.C.); 2 Department of Biotechnology, School of Engineering and Technology (SET), Sharda University, Greater Noida 201310, India; niraj.jha@sharda.ac.in; 3 Department of Pharmaceutical Sciences, Assam University, Silchar 788011, India; itspartha_p@yahoo.com; 4 Department of Botany, University of Calcutta, Kolkata 700019, India; surekha_kundu@yahoo.com; 5 Department of Pharmaceutical Technology, Division of Pharmacognosy, Jadavpur University, Kolkata 700032, India; scm.pharm@jadavpuruniversity.in; 6 Faculty of Biotechnology and Food Sciences, Slovak University of Agriculture in Nitra, 949 76 Nitra, Slovakia; adriana.kolesarova@uniag.sk; 7 Department of Environmental Studies, Institute of Graduate Studies and Research, Alexandria University, Alexandria 21526, Egypt; yousefmokhtar@yahoo.com; 8 Department of Applied Physics, School of Science, Aalto University, 00076 Espoo, Finland; janne.ruokolainen@aalto.fi (J.R.); kavindra.kesari@aalto.fi (K.K.K.); 9 Department of Animal Morphology, Physiology and Genetics, Faculty of AgriSciences, Mendel University in Brno, 61300 Brno, Czech Republic; petr.slama@mendelu.cz

**Keywords:** para-coumaric acid, ROS, oxidative stress, disease, antioxidant, anti-inflammatory, anti-cancer, anti-diabetic, anti-melanogenic

## Abstract

Para-coumaric acid (p-CA) is a plant derived secondary metabolite belonging to the phenolic compounds. It is widely distributed in the plant kingdom and found mainly in fruits, vegetables, and cereals. Various in vivo and in vitro studies have revealed its scavenging and antioxidative properties in the reduction of oxidative stress and inflammatory reactions. This evidence-based review focuses on the protective role of p-CA including its therapeutic potential. p-CA and its conjugates possesses various bioactivities such as antioxidant, anti-inflammatory, anti-cancer, anti-diabetic, and anti-melanogenic properties. Due to its potent free radical scavenging activity, it can mitigate the ill effects of various diseases including arthritis, neurological disorders, and cardio-vascular diseases. Recent studies have revealed that p-CA can ameliorate the harmful effects associated with oxidative stress in the reproductive system, also by inhibiting enzymes linked with erectile function.

## 1. Introduction

Plant secondary metabolites are chemical compounds synthesized through various pathways. Such compounds do not aid in plant growth and development however, they enhance the survival mechanism of plants in different environmental conditions [1]. They possess biological activities and form the parts of human and animal diets. They are predominantly found in fruits, vegetables, cereals, and several other organic food products. Bioactive compounds have great use in disease treatment and management which are utilized as traditional medicine [2]. With a molecular mass of 164.16g/mol, para-coumaric acid (p-CA) is a type of phenolic compound belonging to the hydroxycinnamic acid (HCA) family [3]. It is a class of plant secondary metabolites, which is biologically synthesized through the shikimic acid pathway using phenylalanine and tyrosine as precursors [3,4,5]. It may transform into phenolic acids (e.g., caffeic acid, ferulic acid, chlorogenic acid, and sinapic acid), flavonoids, lignin precursors and other secondary metabolites [3,4]. p-CA is the most abundant isomer of coumaric acid in nature and exists in two forms: cis p-CA and trans p-CA [6]. Compounds belonging to HCA family, such as p-CA are found in green bark of woody vascular plants too [4,7]. Free or bound form of p-CA is widely distributed in fruits (e.g., apple, pear, grape, orange, tomato, and berries), vegetables (e.g., beans, potato, and onion) and cereals (e.g., maize, oat, and wheat) [8]. In such plants, p-CA is found as a component of lignins and tannins [9,10], and is the most prevalent phenolic acids in cereals [11]. p-CA form conjugates with many compounds like alcohols, amines, sugar moiety, lignins, tannins, sterols, hydroxyl acids, etc. Conjugates of p-CA are either water soluble or water insoluble depending on the compounds they are bound with [3]. Water soluble p-CA conjugates are formed when p-CA esterifies with small compounds like alcohols, amines, monosaccharides, etc., while water insoluble p-CA conjugates form when esterified with lignins, tannins, long chain alkyl alcohols or etherified with lignins [12,13]. Conjugates of p-CA can be even more bioactive than free p-CA but these conjugate forms are less likely to be absorbed in the upper gastrointestinal tract [3].

Antioxidant property of p-CA is due to its phenyl hydroxyl group (-OH) that enables them to donate hydrogen or electrons. The median lethal dose (LD50) of p-CA has been found to be 2850 mg/kg in rats [3]. Antioxidant property of p-CA has been associated with the reduction of cadmium-induced renal and liver toxicity [14]. Human studies have suggested that p-CA protects the low-density lipoprotein (LDL) from oxidation and prevents atherosclerosis and coronary heart disease [15]. As a common dietary phenol, p-CA has also been found to inhibit platelet activity both in vitro (in human blood) and in vivo (in rabbit model) [16]. Extracted from the roots of *Aristolochia indica* (*Linn.*), p-CA was reported to inhibit prolactin secretion in female mice and hamsters in vivo [17]. p-CA was also found to reduce serum prolactin level in male mice [18]. p-CA was linked with the reduction of testicular weight, accessory sex organs and acid phosphatase in the prostate gland too. Furthermore, p-CA reportedly decreased sexual desire in adult male rats [19]. A recent study on a rat model reported that p-CA can minimize the reproductive toxicity induced by ethanol. It also enhanced fertility rate by reducing testicular dysfunction induced by ethanol [20]. Another recent in vivo study on rats has reported successful mitigation of the adverse effects of doxorubicin (DOX) by p-CA, which also acts as an aphrodisiac agent in the treatment of erectile dysfunction (ED) [21]. Plant-derived compounds have always been of interest to researchers for the development of new drugs and for the management of various human diseases [22]. p-CA could be a promising candidate for use as a drug against several diseases, particularly characterized by oxidative stress. To understand the dose response of p-CA, both clinical and basic research have been conducted with animal and human models [3,22]. For the benefit of basic scientists, researchers, and clinicians there is a need to review the recent literature in a holistic manner on the possible clinical utility of p-CA.

## 2. Methodology

Search engines such as PubMed, Scopus, and Google Scholar were utilized to collect journal articles regarding the potential use of p-CA in the management of human diseases with a focus on oxidative stress-induced disorders. Keyword strings such as (para-coumaric acid) AND (hydroxycinnamic acid) AND (plant phenolics), (health *) and (management of diseases *), AND (para-coumaric acid) AND (antioxidant), (para-coumaric acid) AND (inflammation) AND (physiology) AND (reproductive physiology) AND (diabetes), (cancer), AND (reproductive health *) ((erection), (sperm), (semen), (ovum), (fertilization)) were used to extract relevant literature from electronic databases. Due to the inability to filter duplicates in the literature search, the number of articles found at each step of the search and filtration process also included duplicates, which were subsequently removed. The conducted search was not restricted to any publication date range. The articles found were carefully reviewed. Article titles were first assessed for relevance, followed by abstracts, and full-text articles. Articles unrelated to this study were removed. Those publications, which did not relate to the specific topics of the current review, were excluded at this stage itself. Exclusion criteria included articles other than in English language.

## 3. Para-Coumaric Acid (p-CA)

In vitro studies have suggested that the consumption of various phenolic compounds could significantly lower the risk of health problems [23]. It is also established that polyphenols suppress the generation of free radicals, as they can reduce the rate of oxidation and deactivate the free radicals [21]. In addition to radical scavenging activity, polyphenols are known metal chelators [15,24]. Phenolic compounds such as HCA can activate antioxidant responsive gene expression (through ARF/Nrf-2 pathway) and protect against severe diseases [25]. HCAs are the major class of natural phenolic compounds, having a C6-C3 carbon skeleton with a double bond in the side chain that may have *cis* or *trans* configuration [26]. Common HCAs include cinnamic acid, o-coumaric acid, m-coumaric acid, p-coumaric acid, caffeic acid, ferulic acid and sinapic acid [4]. They play a scavenging role against oxidative stress, where these are found as free carboxylic acids, esters, or amides. p-CA is one of the most important HCAs, as it plays a key role in secondary metabolism, which may transform into phenolic acids (e.g., caffeic acid, ferulic acid, chlorogenic acid, and sinapic acid), flavonoids, lignin precursors and other secondary metabolites [3,4]. p-CA exhibits various biological properties namely antioxidant, anti-inflammatory, and anti-cancer agents. With its antioxidant and anti-inflammatory properties, p-CA can mitigate the harmful effects of various diseases such as diabetes and rheumatoid arthritis.

### 3.1. Alleviation of Oxidative Stress

Several studies suggested that p-CA can reduce oxidative stress due to its scavenging property against free radical formation [27,28,29]. The antioxidant property of p-CA is attributed to its phenyl hydroxyl group (-OH) that enables it to donate hydrogen or electrons. The antioxidant activity of p-CA has been demonstrated in several experiments, where it showed protective effects on cultured endothelial cells against oxidative stress induced by high glucose and free fatty acids [30]. p-CA can also minimize the oxidative stress in keratinocytes exposed to ultraviolet radiation [31]. In lens epithelial cells, p-CA has been shown to mitigate the effects of oxidative stress induced by hydrogen peroxide (H_2_O_2_) [32].

In vivo studies on a rat model suggested that p-CA at 50 mg/kg body weight can reduce basal oxidative DNA damage more effectively than vitamin E and induce glutathione (GSH) like enzymes such as glutathione S-transferase Mu 2(GST-M2) in colonic mucosa [33]. Another study on a rat model reported that p-CA at a dose of 8 mg/kg body weight minimizes the adverse effects of isoproterenol in myocardium. A high dose of isoproterenol (100 mg/kg body weight) resulted in acute myocardial necrosis by generating free radicals. Although, free radical scavenging activity of p-CA prevents cardiac damage induced by isoproterenol [29]. Cardio-protective effects of p-CA was revealed by a reduction in lipid peroxidation level, cholesterol oxidation, and low-density lipoprotein (LDL) resistance [34,35]. An in vitro study in PC12 cells and another study in mice reported p-CA to exhibit anti-hyperlipidemic property, where it reduced the oxidative stress against high lipid diet, and protected liver from steatosis [36]. In the rat model, p-CA was also found to reduce LDL oxidation [15].

The protective role of p-CA on renal tissue was reported as it reduced oxidative stress and minimized cadmium-induced renal toxicity in albino Wistar rats [14]. In mice, at a dose of 100 mg/kg body weight, p-CA increased neuronal strength against ischemia reperfusion injury by reducing oxidative stress and increasing superoxide dismutase (SOD) and catalase (CAT) activities [37]. Another in vivo study reported the neuroprotective effects of p-CA against cisplatin, an anti-neoplastic drug possessing neurotoxic effects by producing oxidative stress; pretreatment of rats with p-CA at a dose of 100 mg/kg body weight reduced oxidative stress by enhancing SOD and GSH activity and minimizing the effects of cisplatin [38]. Moreover, the antioxidant property of p-CA enhanced upon conjugation with quinic acid, monosaccharides, and amines [3,39]. An in vivo study in rats has reported that p-CA at doses of 50, 100 and 200 mg/kg body weight can minimize the reproductive toxicity induced by ethanol. It also restored male fertility by reducing testicular dysfunction induced by ethanol through its antioxidant property [20]. Another recent in vivo study in a rat model suggested that at doses of 50 and 100 mg/kg body weight p-CA inhibits the erectogenic enzymes associated with ED. It further improves non-protein thiol (an antioxidant) level in the penile tissue and acts as an aphrodisiac agent against doxorubicin (DOX)-induced ED. DOX induced penile tissue showed higher levels of erectogenic enzymes, however, treatment with p-CA reduced the level of erectogenic enzymes and mitigated the adverse effects of DOX in male rats [21].

### 3.2. Mitigation of Inflammation

p-CA has an anti-inflammatory activity as it decreases the expression of the inflammatory mediators such as TNF-α and IL-6. Nuclear factor kappa light chain enhancer of activated B cells (NF-κB) is a family of transcription factor that regulates inflammatory response i.e., the production of cytokines. Studies have shown that p-CA can inhibit the activation of NF-κB and reduce the levels of TNF-α [40,41,42].

In rats, at a dose of 100 mg/kg body weight, p-CA reduced the effects of monosodium urate (MSU) crystals by its anti-inflammatory activity in vivo. MSU triggers inflammatory reaction by enhancing the release of cytokines that result into a disease condition—gout. p-CA, by virtue of its antioxidant and anti-inflammatory activities, reduced the expression of cytokines [40]. In lipopolysaccharide (LPS) induced sepsis rat model, p-CA showed antioxidant and anti-inflammatory effects in vivo. At 100 mg/kg body weight, it reduced the levels of pro-inflammatory cytokines (TNF-α, IL-1β, IL-6) in lungs and liver, and further increased the levels of anti-inflammatory cytokines (IL-4, IL-10), when treated in combination with ellagic acid (EA). In LPS induced septic rats, p-CA also increased the antioxidant levels [43]. p-CA at doses of 50, 100 and 200 mg/kg body weight in rats, alleviates ethanol induced nephrotoxicity by inhibiting NF-kB signaling which reduces the pro-inflammatory cytokines production, it also lowers the cellular apoptosis by elevating Bcl-2 expression and restores antioxidant level [42].

### 3.3. Role in Cancer

p-CA and ferulic acids (FA) at a dose of 150 μM have been able to change gene expression in human Caco-2 colon cancer cell lines and caused changes in cell cycle progression. p-CA impairs the G2/M phase of the cell cycle too. It also possesses anti-proliferative activity, and further inhibited Caco-2 and ECV304 cells at high concentrations of 1.5 and 5 mmol/L [44].

Furthermore, p-CA has been shown to moderately inhibit the growth of some tumor cell lines and decrease the viability of neuroblastoma cells N2a (EC50 = 104 μmol/L), human lung cells A549, and colon cells HT29-D4, by reducing the adhesion and migration of cancer cells and helping with inhibition of spread of cancer cells and cancer stem cells [44,45,46,47].

4-O-(2″-O-Acetyl-6″-O-pcoumaroyl-D-glucopyranosyl)-p-CA, a conjugate of p-CA, isolated from *Bidens pilosa* demonstrated high anti-proliferative activity in lung cancer cell lines A549, NCI-H1299 and HCC827 with IC50 values of 37.73, 50.6 and 62.0 μg/mL, respectively [48]. Kaempferol-3-(6″-coumaroyl glucoside), another conjugate of p-CA, isolated from *Solanum elaeagnifolium* also showed high cytotoxic effect against breast cancer cell line MCF7 (IC50 = 6.9 μmol/L), and the liver cancer cell line HPG2 (IC50 = 32.6 μmol/L) [3,48]. These conjugates of p-CA showed anti-tumor activity by elevating the action of proteins involved in the mitochondria-mediated apoptosis (i.e., C-caspase-3, C-caspase-9, Bcl-2, Bcl-xl, Bcl-xs, Mcl-1, Bad and Bax) [12]. An in vitro study involving ECV304 cell line reported that p-CA inhibits the AKT and ERK signaling pathways thus hindering cell proliferation, these signaling pathways are followed by cancer cells [49].

### 3.4. Role in Diabetes

Studies also reported the anti-diabetic activity of p-CA where, at 100 mg/kg body weight, blood glucose levels declined and blood insulin levels elevated in streptozotocin (STZ)-induced diabetic rats. STZ causes devastation of pancreatic β cells, and results in hyperglycemia. STZ-induced diabetic rats also showed lower levels of vitamins E, C, GSH and SOD, but pre-treatment with p-CA presented with normal blood levels of insulin, vitamins E and C, GSH and SOD [50]. Another study reported that p-CA through its antioxidant properties can modulate the gluconeogenic enzymes and help in lowering of the blood glucose level. At a dose of 100 mg/kg body weight administered on STZ-induced diabetic rats, p-CA also increased the hexokinase activity as well as the expression of glucose transporter 2 (GLUT2) mRNA in pancreas [51].

A recent in vivo study in a rat model suggested that p-CA exhibits a protective role against diabetic nephropathy (DN) through its anti-inflammatory and antioxidant properties [52]. DN rats showed high levels of TLR 4, IL-6, and TGF β1 whereas treatment with p-CA at a dose of 100 mg/kg body weight reduced the levels of TLR 4, IL-6, and TGF β1. Via its antioxidant property, p-CA also reduces the level of MDA and increases the level of SOD [52]. A study revealed that p-CA derivatives inhibit alpha-glucosidase activity, and alpha-glucosidase results in delayed digestion of starch and sucrose into glucose, and hence lowers the blood glucose level [53].

p-CA increases the phosphorylation of AMP activated protein kinase (AMPK) in L6 skeletal muscle cells, where this may result in modulation of glucose and lipid metabolism. AMPK is an enzyme that regulates energy homeostasis by boosting glucose uptake [54]. p-CA also increases phosphorylation of acetyl CoA carboxylase (ACC) as well as the expression of carnitine palmitoyltransferase-1 (CPT-mRNA) and peroxisome proliferator-activated receptor (PRAR) that may result in elevated beta–oxidation of fatty acids, and triacylglycerol synthesis [54]. An in vivo study in rat liver suggested that p-CA can inhibit gluconeogenesis. At IC50 value 92.5 μmol/L, p-CA inhibited the transformation of lactose into glucose, whereas at IC50 value of 75.7 μmol/L it inhibited the production of glucose from alanine and ultimately resulted in lowered blood glucose level [55].

### 3.5. Role in Dermatology

Several studies reported the anti-melanogenic effect of p-CA. Isolated from *Sasaquel paertensis*, culms and leaf extract containing p-CA inhibited melanogenesis in murine melanoma B16/F10 cells stimulated with alpha-melanocyte (α-MSH) stimulating hormone [56]. It also showed anti-melanogenic effects on human melanocytes [56]. Melanin is synthesized from tyrosine, catalyzed by tyrosinase (TYR) enzyme, and p-CA has close structural similarity with tyrosine [57,58]. p-CA due to structural similarity with tyrosine, is impaired with mushroom tyrosinase (TYR) enzyme, and inhibits tyrosine oxidation [59]. p-CA has been shown to possess a strong capacity to inhibit human TYR, and it also mitigated ultraviolet (UV) radiation toxicity in human skin cells in vitro [60]. Ex vivo skin permeation experiment and in vivo testing suggested that p-CA in the form of cream can diffuse into skin. Additionally, methyl p-CA also possesses anti-melanogenic effects [61]. A recent study suggested that p-CA acts as skin lightening agent and can be used in cosmetics [58]. Another in vitro study in HaCaT cells reported that p-CA possesses an anti-photoaging effect and was found to minimize the effects of UVB induced stratifin release from keratinocytes, which resulted in mitigation of matrix metalloproteinase 1 (MMP1). Stratifin-induced MMP1 expression in fibroblast resulted in skin remodeling and formation of wrinkles, and p-CA reportedly acts as an anti-photoaging agent via attenuated stratifin release [31]. The various in vivo and in vitro studies have been depicted in Table 1 and Table 2.

## 4. Mechanism of Action

p-CA mitigates the effects of several diseases by modulating various mechanisms. Several studies suggested that p-CA elevates the level or the activity of enzymes that reduce oxidative stress and inflammation [2,36,52]. Several factors and/or mechanisms through which p-CA exerts its beneficial effects are discussed below.

### 4.1. Antioxidative Mechanism

Every living organism maintains a condition of homeostasis between the oxidative stress and antioxidant species. Oxidative stress refers to the excessive production of reactive oxygen species (ROS) in the cells and tissues. ROS are normally produced in minimal quantity and are involved in the regulation of processes such as signal transduction, gene expression, and activation of receptors [66]. Oxidative stress can damage the cellular structures, such as cell membrane, lipids, proteins, and DNA [67]. However, the body’s antioxidant systems counteract the effects of ROS. Imbalance in this process can lead to the damage of cellular molecules including DNA, proteins, and lipids [68]. Furthermore, uncontrolled oxidative stress can accelerate the aging process and may contribute to the development of a number of diseases. Oxidative stress is induced by free radicals such as superoxide radicals (O_2_^−^), hydrogen peroxide (H_2_O_2_), hydroxyl radicals (OH), and singlet oxygen (^1^O_2_) which are commonly defined as ROS [69]. The major site of generation of ROS is mitochondria under both physiological and pathological conditions [70].

**Figure 1:**
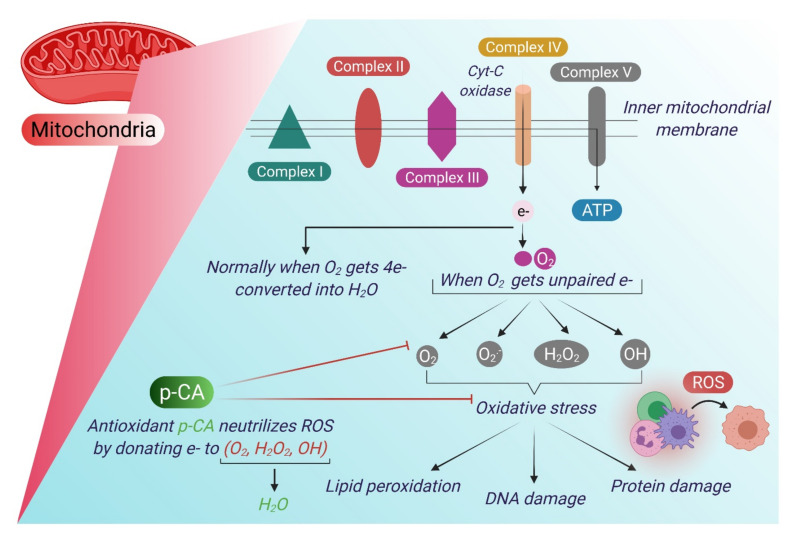
Generation of oxidative stress and its neutralization by p-CA. Oxidative phosphorylation takes place in the inner mitochondrial membrane that results in the synthesis of ATP. During oxidative phosphorylation, electrons flow from complex I to complex IV and finally, are accepted by O_2_. Normally, O_2_ accepts 4e- and gets converted into H_2_O but when it accepts unpaired electrons it generates ROS. p-CA can neutralize ROS by donating e- to ROS (such as O_2_, H_2_O_2_, OH, ^1^O_2_) and produces H_2_O.

**Figure 2:**
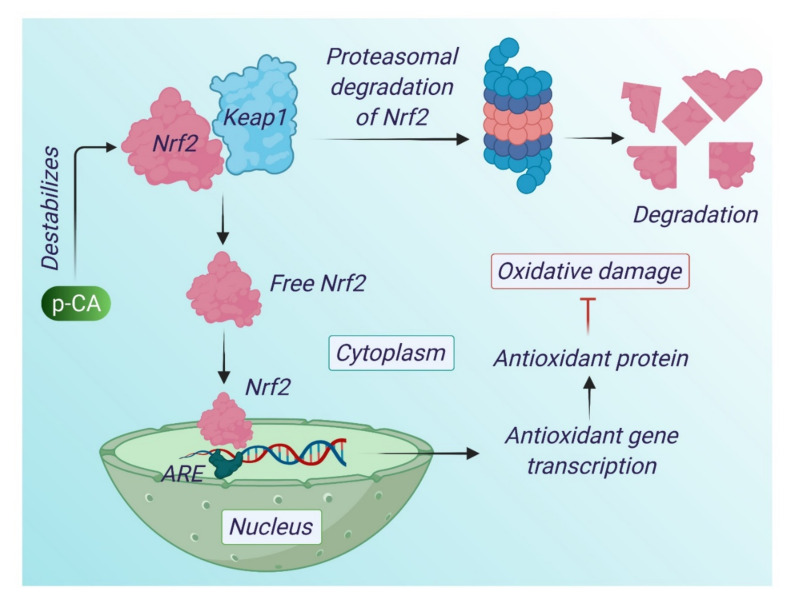
Action of p-CA in ARE/Nrf2 pathway. Nuclear factor erythroid 2-related factor 2 (Nrf2) is a transcription factor of the leucine zipper family, which regulates the expression of antioxidant enzymes/proteins. It has a specific repressor in the cytoplasm namely Keap1 (Kelch-like ECH-associated protein 1), which mediates the proteosomal degradation of Nrf2. Oxidative stress conditions, and many exogenous chemicals, alter the redox status of keap1 cysteine residues that result in destabilization of keap1, which allows Nrf2 translocation to the nucleus. In the nucleus it binds with antioxidant response element (ARE) resulting in the synthesis of antioxidant enzymes. p-CA can destabilize the keap1 and this results in the activation of Nrf2 and helps in the synthesis of antioxidant enzymes.

HCAs such as p-CA, FA and CA have been found to activate the expression of antioxidant responsive genes via the ARF/Nrf-2 pathway [25]. An in vivo study in a rat model suggested that p-CA can activate nuclear factor erythroid 2 related factor (Nrf2), a transcription factor that regulates antioxidant response element (ARE)-mediated gene expression of downstream target genes, such as glutathione peroxidase that elevate the cardiac antioxidant capacity. The antioxidative mechanisms of p-CA are depicted in Figure 1 and Figure 2.

### 4.2. Anti-Inflammatory Mechanism

**Figure 3:**
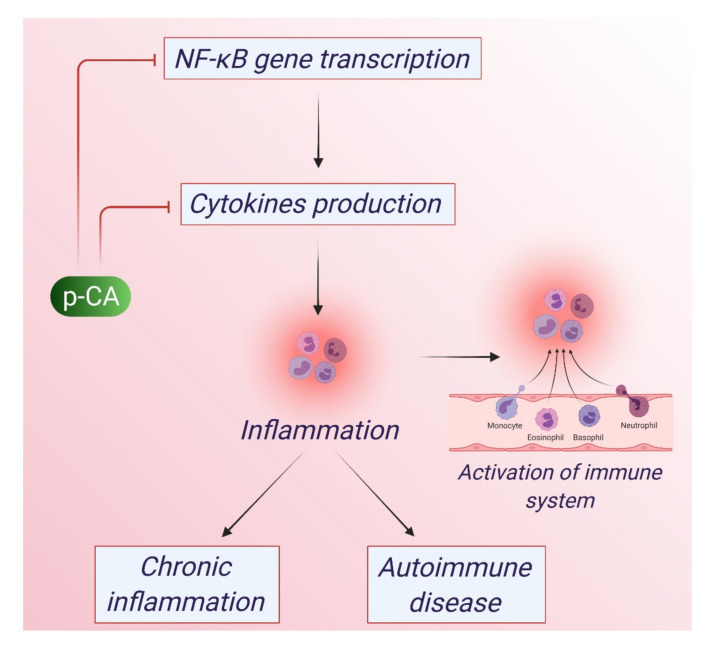
Anti-inflammatory action of p-CA. p-CA impairs the function of NF-kB and reduces the production of cytokines. The products of NF-kB genes act as transcription factors of pro-inflammatory cytokines. p-CA modulates the function of NF-kB gene and inhibits its downstream pathways thereby reducing the cytokine production. By inhibiting NF-kB gene, p-CA acts as an anti-inflammatory agent.

Inflammation is a vital part of the immune system’s response to injury and infection [71]. It is a way of signaling the immune system to heal and repair the damaged tissue as well as defend itself against foreign invaders, such as viruses and bacteria [72]. However, excessive prolonged inflammatory response can be problematic. Chronic inflammation has been linked to certain diseases, such as heart disease, cancer, Alzheimer’s disease, rheumatoid arthritis, and diabetes [73]. However, a healthy diet and lifestyle can help to keep inflammation under control. NF-_K_B is an inducible transcription factor, and after its activation many genes that result in the production of cytokines possibly get activated, thereby regulating inflammation [71]. However, prolonged inflammation can lead to many chronic diseases, and p-CA can mitigate such inflammation through its anti-inflammatory action [40,41]. The anti-inflammatory mechanism of p-CA is depicted in Figure 3.

### 4.3. Anti-Cancer Mechanism

**Figure 4:**
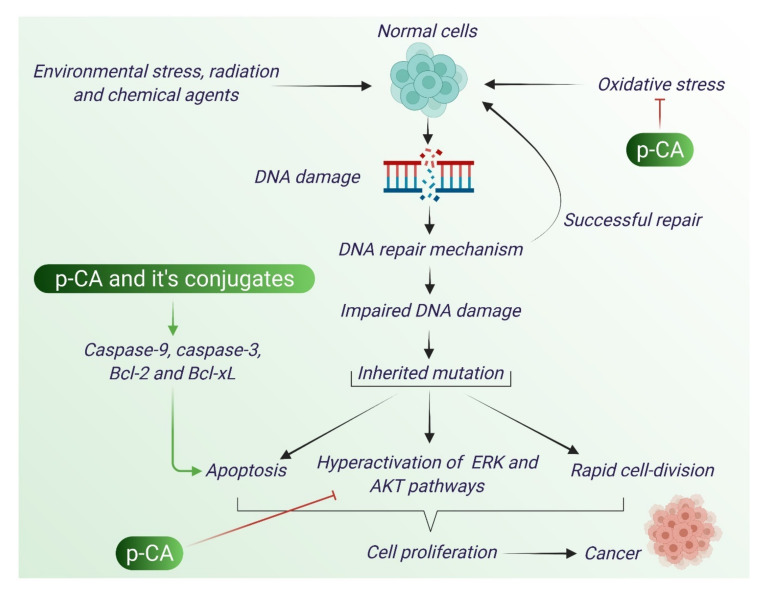
Anti-cancer action of p-CA. p-CA acts an anti-cancer agent through impairment of a number of mechanisms that promote cancer. By modulating ERK and AKT pathways p-CA can inhibit cell proliferation. It also activates pro-apoptotic factors and induces apoptosis in cancer cells.

Cancer is a group of diseases that results from uncontrolled cell division. Carcinogenesis can develop in a number of ways including rapid self-proliferation, insensitivity to anti-proliferative signals, and escaping signaling from apoptosis. The mechanism of action of anti-cancer drugs can be varied such as by affecting the gene that regulates cell proliferation, cell cycle, apoptosis; or by suppressing the enzymes that are needed for cell proliferation [46]. ROS enhance initiation of tumor formation and lead to carcinogenesis [44,45,46]. The anti-cancer mechanism of p-CA is depicted in Figure 4.

### 4.4. Anti-Diabetic Mechanism

**Figure 5:**
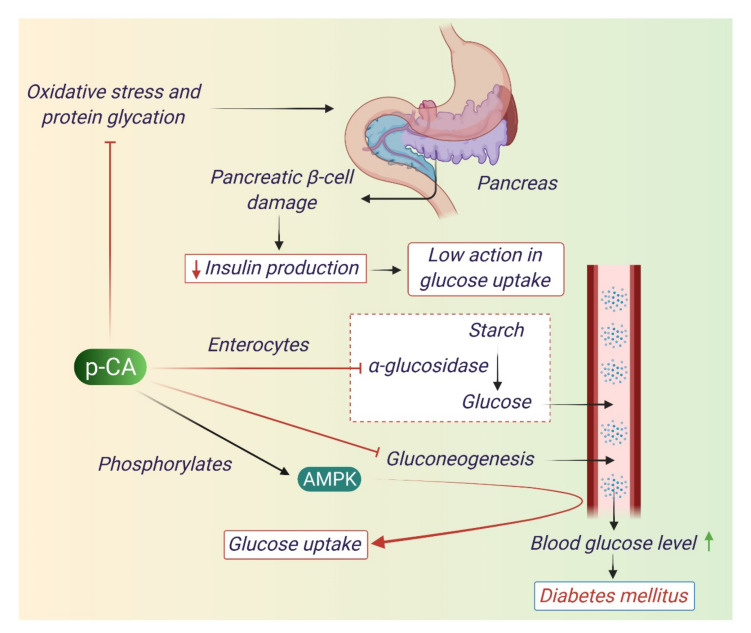
Antidiabetic mechanism of p-CA. p-CA mitigates diabetes mellitus through a number of mechanisms. Diabetes mellitus is characterized by high blood glucose. p-CA acts as an anti-diabetic agent by reducing blood glucose level. It reduces blood glucose level by inhibiting gluconogenesis, a mechanism where non-carbohydrates (proteins and fatty acids) are converted into glucose in the liver. p-CA also protects pancreatic beta cells from damage by minimizing oxidative stress in the pancreas. It also reduces the glucose absorption in blood by inhibiting alpha glucosidase enzyme which is necessary for converting starch to glucose. By modulating AMPK enzymes, p-CA can increase the uptake of glucose.

Diabetes mellitus (DM) is the body’s inability to regulate the level of glucose in the blood. Chronic hyperglycemia may occur due to abnormal insulin secretion or action and may further lead to disorders in the metabolism of carbohydrates, fats, and proteins [74]. During diabetes mellitus, ROS production increases via auto-oxidation of glucose and non-enzymatic protein glycation which may also result in cellular oxidative damage [50]. p-CA reduces oxidative stress by enhancing the production of antioxidant enzymes and attenuates the oxidative stress induced by DM [75]. Insulin is a hormone secreted by pancreatic beta cells maintains glucose homeostasis by regulating the actions of different enzymes. Uncontrolled hyperglycemia may cause several pathological conditions such as neuropathy, nephropathy, and retinopathy [76]. In muscles and adipose tissue, glucose uptake is controlled by the action of insulin pathway [77]. p-CA increases the insulin production and GLUT 2 expression [51]. p-CA reduces the actions of gluconeogenic enzymes and enhances the activity of hexokinase, glucose 6 phosphatase dehydrogenase and helps in lowering blood glucose level [51,55]. p-CA acts as an AMPK pathway activator and through which it helps to reduce blood glucose level [54]. The anti-diabetic mechanism of p-CA is depicted in Figure 5.

### 4.5. Anti-Melanogenic Activation and Mechanism of Action

**Figure 6:**
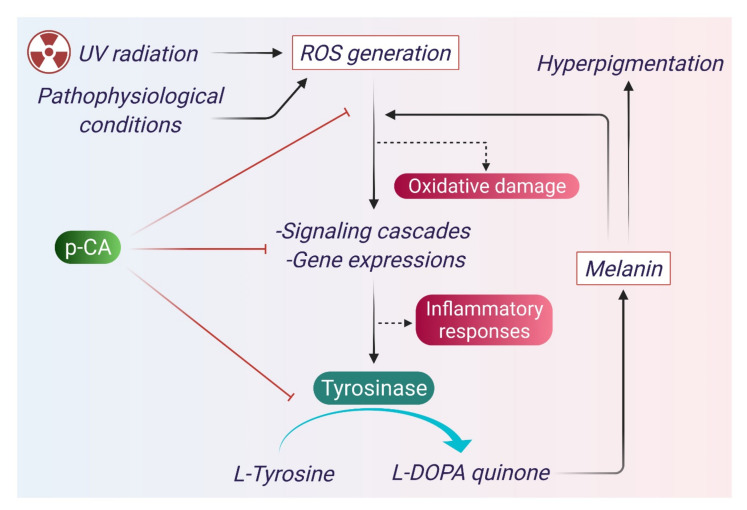
Melanin is synthesized in the melanocytes catalyzed by specific enzymes. UV induced oxidative stress in melanocytes results in activation of different genes that lead to elevated expression of tyrosinase. Tyrosine is the precursor of melanin; it first converts into L-DOPA quinine through the action of tyrosinase. L-DOPA quinine then converts into melanin by a series of enzymes. p-CA has structural similarity with tyrosine and competes with tyrosine for the same enzyme tyrosinase and thus impairs melanin synthesis. p-CA also reduces UV induced oxidative stress and impairs signaling pathways that lead to gene expression for tyrosinase synthesis.

UV radiation incites DNA damage and acts as a major factor for carcinoma. It also causes skin damage, and its repair system induces melanogenesis [78]. Melanin, a natural skin pigment has beneficial effects on the skin via prevention of UV radiation-induced skin damage [79]. Abnormal accumulation of melanin may result in hyperpigmentation [58]. Melanin is synthesized in melanocytes with the help of the enzyme tyrosinase. p-CA acts as an anti-melanogenic agent by impairing the melanin synthesis pathway [56,57,61]. By reducing melanin concentration in skin cells, p-CA acts as a hypo-pigmenting agent and has attracted special interest as an ingredient in cosmetic products [58]. p-CA acts as a skin hypo-pigmenting agent via multiple mechanisms [31]. UV radiation stimulates ROS generation in epithelial cells of skin, which induces several pathways and results in generation of melanin with the help of tyrosinase enzyme [79,80]. Tyrosinase plays a central role in the biosynthesis of melanin, and melanin again absorbs UV radiation and reduces oxidative stress [81]. Melanin deposition in the skin results in darker skin complexion. p-CA has structural similarity with tyrosine. It competes with tyrosine for the same enzyme tyrosinase and impairs melanin synthesis by inhibiting the tyrosinase enzyme [57]. p-CA also reduces ROS production through its antioxidant activity and reduces oxidative stress [58]. The anti-melanogenic mechanism of p-CA is depicted in Figure 6.

## 5. Conclusions and Future Perspectives

Plant parts have been used for the control of various diseases since ancient times. In the past few years, phytochemicals have gained renewed attention for discovery of drugs for the treatment of various diseases. As a large number of drugs have been developed from phytochemicals either directly or indirectly, the knowledge of chemical structure and mechanisms of action of phytochemicals can be used further to develop p-CA based drugs for the management of several oxidative stress induced diseases [82].

Belonging to the HCA family of phytochemicals, p-CA is synthesized from the precursor tyrosine and phenylalanine through the shikimic acid pathway. It is abundant in many edible plants and their fruits. Presence of ^–^OH group in its structure enables donation of H+ to free radicals and neutralizes them [26,83]. Thus, p-CA possesses radical scavenging property and can mitigate the diseased condition caused by ROS and basal DNA oxidative damage. Various in vivo and in vitro studies have shown p-CA as a powerful antioxidant, anti-diabetic, anti-cancer, and anti-inflammatory agent [83]. Studies reported that p-CA enhances the production of antioxidant enzymes by activating the ARF/Nrf-2 pathway [25]. p-CA can also interfere with the NF-κB pathway and reduce the production of pro-inflammatory cytokines thus minimizing the disease condition caused by hyper-inflammation [40,41,42]. Through its antioxidant and anti-inflammatory activities, p-CA also mitigates the adverse effects of arthritis, cancer, diabetes and several other neurological and nephrological disorders [3]. It also acts as a cardioprotective, UV protective and skin-lightening agent. By possessing structural similarity with tyrosine, p-CA can impair the tyrosinase enzyme and lower the rate of melanin synthesis and acts as a hypo-pigmenting agent [58,59,60]. p-CA protects different organs from oxidative damage and inflammation. Oxidative stress also affects the reproductive system of the body and reduces fertility, and p-CA through its antioxidant property may be able to mitigate such adverse effects of oxidative stress in reproductive tissues. It reduces erectogenic enzymes such as arginase, phosphodiesterase (PDE)-5, AchE and AMPdase and ameliorates ED [21].

At doses of 50 and 100 mg/kg body weight, p-CA exhibited advantageous effects against various oxidative stress-induced diseases in animal models. By calculating these animal doses of p-CA in humans, it may be proposed that p-CA at a dose range of 8.11–16.22 mg/kg body weight may give beneficial clinical results in humans. However, before any therapeutic use, these doses must be validated through human experimentations and clinical trials.

## Figures and Tables

**Table 1:** Clinical significance of p-CA. In vivo dose responses of p-CA administration in various experimental models are shown below.

Experimental Model	Dose	Results	Calculated Human Dose [62]	Reference(s)
Rat	100 mg/kg body weight	Protective effects on diabetic neuropathy (DN) through anti-inflammatory activity	16.22 mg/kg body weight	[52]
Mouse	100 mg/kg body weight	Anti-hyperlipidemic activity by reducing lipid aggregation in liver tissue in high fat diet	8.11 mg/kg body weight	[36]
Rat	100 mg/kg body weight	Reduction of oxidative stress on penile tissue induced by doxorubicin (DOX); mitigation of adverse effects of erectogenic enzymes and acting as an aphrodisiac agent in the treatment of erectile dysfunction (ED) in the male	16.22 mg/kg body weight	[21]
Rat	100 mg/kg body weight	Protective effects against lipopolysaccharides (LPS)-induced lung inflammation	16.22 mg/kg body weight	[63]
Rat	100 mg/kg body weight	Protective effects on rheumatoid arthritis (RA) by suppression of inflammatory cytokines	16.22 mg/kg body weight	[64]
Rat	50, 100 and 200 mg/kg body weight	Reduction of stress on testicular tissue induced by ethanol and improvement of reproductive health in the male	8.11, 16.22 and 32.43 mg/kg body weight	[20]
Mouse	100 mg/kg body weight	Neuroprotective effects by reducing ischemia reperfusion (IR)-induced brain oxidative stress	8.11 mg/kg body weight	[37]
Rat	100 mg/kg body weight	p-CA reduced the neurotoxicity in cisplantin-induced animals through its antioxidant property	16.22 mg/kg body weight	[38]
Rat	100 mg/kg body weight	Acting as an anti-diabetic agent, p-CA lowers the action of gluconeogenic enzymes and enhances the expression of glucose transporter 2 (GLUT 2) in pancreatic beta cells	16.22 mg/kg body weight	[51]
Rat	100 mg/kg body weight	Protective effects against cadmium chloride-induced renal toxicity through its antioxidant property and metal chelating activity	16.22 mg/kg body weight	[14]
Rat	8 mg/kg body weight	Minimizes the effect of isoproterenol on myocardium by maintaining lysosomal lipid peroxidation level	1.3 mg/kg body weight	[29]
Rat	100 mg/kg body weight	Mitigates the effects of gout via anti-inflammatory activity against monosodium urate (MSU) crystals-induced inflammation	16.22 mg/kg body weight	[40]
Rat	50 and 100 mg/kg body weight	Protective effects on sepsis through antioxidant and anti-inflammatory property	8.11 and 16.22 mg/kg body weight	[43]
Rat	50 mg/kg body weight	Reduction of basal DNA damage in rat colonic mucosa cells by increasing antioxidant enzyme levels	8.11 mg/kg body weight	[33]

**Table 2:** Clinical significance of p-CA. In vitro dose responses of p-CA administration in various experimental models are shown below.

Experimental Model	Dose	Results	Reference(s)
Bovine aorticendothelial cells (BAEC)	20, 40, 80 and 160 mM	Antioxidant property in endothelial cells exposed to high glucose	[30]
Human keratinocyte cell line (HaCaT)	0.018, 0.06 and 0.18 mM	As an anti-photoaging agent, p-CA reduces the expression of UV induced stratifin in keratinocytes	[31]
Rat pheochromocytoma cell linePC12	0.30mM	Protective effects on PC12 cells against 2,2′-Azobis(2-amidinopropane), dihydrochloride (AAPH) induced oxidative stress; p-CA reduced the level of malondialdehyde (MDA) and lactate dehydrogenase (LDA) production in AAPH induced PC12 cells and acted as antioxidant and anti-hyperlipidemic agents	[36]
Murine macrophage cell lineRAW264.7	0.06, 0.30 and 0.60 mM	Inhibition of pro-inflammatory cytokine production by blocking NF-kB and MAPK pathways; protective effects against LPS induced RAW264.7 cells	[41]
A549 cells	20 mM	p-CA shows protective effects on LPS induced lung inflammation in rat and A549 cells	[63]
Human colonic cell line Coca-2	1.5 mM	Inhibition of cell proliferation by affecting cell cycle	[44]
Mouse neuroblastoma cell lineN2a	0.15 mM	Acts as an anti-cancer agent in N2a neuroblastoma cells by inducing apoptosis	[47]
Rat endothelial ECV304 cells	0.5, 1, 2.5 and 5mM	Inhibition of angiogenesis and tumor growth at 5 mM dose	[49]
Colon cancer lines HCT-15 and HT-29	1.4 mM (HCT-15) and 1.6 mM (HT-29)	p-CA acts as anti-cancer agent by inducing mitochondrial mediated apoptosis	[65]
Lung cancer cell lines A549, NCI-H1299 and HCC827	0.06, 0.12, 0.24, 0.36, 0.48 and 0.61 mM	p-CA conjugates induced apoptosis by upregulation of caspase-3, caspase-9, bax, bad and downregulation of Bcl-2	[3]
Rat liver	0.05, 0.1, 0.2, 0.25 and 0.3 mM	Inhibition of gluconeogenesis from lactate and alanine, thus lowering blood glucose level	[55]
L6 skeletal muscle cells	0.1 mM	Modulation of AMPK, increase of glucose uptake, enhancement of fatty acid beta oxidation, and prevention of type-2 diabetes	[54]
Murine melanoma cell line B16/F10 and human melanocytes	0.1 mM	Inhibition of cellular melanogenesis in both B16/F10 and human melanocytes by attenuating a-MSH and tyrosinase expression	[56]
Ex vivo mouse model	0.1 mM	Inhibition of tyrosinase enzyme; inhibition of melanin biosynthesis	[58]

## References

[1] Jain C., Khatana S., Vijayvergia R. (2019). Bioactivity of secondary metabolites of various plants: A review. Int. J. Pharm. Sci. Res..

[2] Hussein R.A., El-Anssary A.A. (2019). Plants secondary metabolites: The key drivers of the pharmacological actions of medicinal plants. Herb. Med..

[3] Pei K., Ou J., Huang J., Ou S. (2016). p-Coumaric acid and its conjugates: Dietary sources, pharmacokinetic properties and biological activities. J. Sci. Food Agric..

[4] El-Seedi H.R., El-Said A.M.A., Khalifa S.A.M., Göransson U., Bohlin L., Borg-Karlson A.-K., Verpoorte R. (2012). Biosynthesis, natural sources, dietary intake, pharmacokinetic properties, and biological activities of hydroxycinnamic acids. J. Agric. Food Chem..

[5] Herrmann K.M., Weaver L.M. (1999). The shikimate pathway. Annu. Rev. Plant. Biol..

[6] Kort R., Vonk H., Xu X., Hoff W.D., Crielaard W., Hellingwerf K.J. (1996). Evidence for trans-cis isomerization of the p-coumaric acid chromophore as the photochemical basis of the photocycle of photoactive yellow protein. FEBS Lett..

[7] Tanase C., Coșarcă S., Muntean D.L. (2019). A critical review of phenolic compounds extracted from the bark of woody vascular plants and their potential biological activity. Molecules.

[8] Alamed J., Chaiyasit W., McClements D., Decker E.A. (2009). Relationships between free radical scavenging and antioxidant activity in foods. J. Agric. Food Chem..

[9] Barros L., Dueñas M., Ferreira I.C., Baptista P., Santos-Buelga C. (2009). Phenolic acids determination by HPLC–DAD–ESI/MS in sixteen different Portuguese wild mushrooms species. Food Chem. Toxicol..

[10] Krishna N.A.V., Nadeem M.D., Saradhi M.P., Mahendran B., Bharathi S. (2014). Cumulative activity of the p-coumaric acid and syringaldehyde for antimicrobial activity of different microbial strains. Euro J. Exp. Biol..

[11] Hole A.S., Grimmer S., Jensen M.R., Sahlstrøm S. (2012). Synergistic and suppressive effects of dietary phenolic acids and other phytochemicals from cereal extracts on nuclear factor kappa B activity. Food Chem..

[12] Sun R.C., Sun X.F., Zhang S.H. (2001). Quantitative determination of hydroxycinnamic acids in wheat, rice, rye, and barley straws, maizestems, oil palm frond fiber, and fast-growing poplar wood. J. Agric. Food Chem..

[13] Xu F., Sun R.C., Sun J.X., Liu C.F., He H.B., Fan J.S. (2005). Determination of cell wall ferulic and p-coumaric acids in sugarcane bagasse. Anal. Chim. Acta.

[14] Navaneethan D., Rasool M.K. (2014). An experimental study to investigate the impact of p-coumaric acid, a common dietary polyphenol, on cadmium chloride-induced renal toxicity. Food Funct..

[15] Zang L.Y., Cosma G., Gardner H., Shi X., Castranova V., Vallyathan V. (2000). Effect of antioxidant protection by p-coumaric acid on low-density lipoproteincholesterol oxidation. Am. J. Physiol. Cell Physiol..

[16] Luceri C., Giannini L., Lodovici M., Antonucci E., Abbate R., Masini E., Dolara P. (2007). p-Coumaric acid, a common dietary phenol, inhibits platelet activity in vitro and in vivo. Br. J. Nutr..

[17] Pakrashi A., Pakrasi P. (1979). Antifertility efficacy of the plant *Aristolochia indica* Linn on mouse. Contraception.

[18] Chowdhury M., Kabir S.N., Pal A.K., Pakrashi A. (1983). Modulation of luteinizing hormone receptors: Effect of an inhibitor of prolactin secretion, p-coumaric acid. J. Endocrinol..

[19] Pakrashi A., Kabir S., Ray H. (1981). 3-(4-Hydroxy phenyl)-2-propenoic acid—A reproductive inhibitor in male rat. Contraception.

[20] Nishi K., Ramakrishnan S., Gunasekaran V.P., Parkash K., Ramakrishnan A., Vijayakumar N., Ganeshan M. (2018). Protective effects of p-coumaric acid on ethanol induced male reproductive toxicity. Life Sci..

[21] Oyeleye S.I., Adefegha S.A., Dada F.A., Okeke B.M., Oboh G. (2019). Effect of p-coumaric acid on the erectogenic enzyme activities and non-protein thiol level in thepenile tissue of normal and doxorubicin-induced oxidative stress male rat. Andrologia.

[22] Kesari K.K., Dhasmana A., Shandilya S., Prabhakar N., Shaukat A., Dou J., Rosenholm J.M., Vuorinen T., Ruokolainen J. (2020). Plant-derived natural biomolecule picein attenuates menadione induced oxidative stress on neuro blastoma cell mitochondria. Antioxidants.

[23] Lou Z., Wang H., Rao S., Sun J., Ma C., Li J. (2012). p-Coumaric acid kills bacteria through dual damage mechanisms. Food Control..

[24] Tsao R. (2010). Chemistry and biochemistry of dietary polyphenols. Nutrients.

[25] Rodrigo R., Miranda A., Vergara L. (2011). Modulation of endogenous antioxidant system by wine polyphenols in human disease. Clin. Chim. Acta.

[26] Teixeira J.C.S., Gaspar A., Garrido E.M., Garrido J., Borges F. (2013). Hydroxycinnamic acid antioxidants: An electrochemical overview. BioMed Res. Int..

[27] Ferguson L.R., Zhu S.-T., Harris P.J. (2005). Antioxidant and antigenotoxic effects of plant cell wall hydroxycinnamic acids in cultured HT-29 cells. Mol. Nutr. Food Res..

[28] Gani A., Wani S.M., Masoodi F.A., Hameed G. (2012). Whole-grain cereal bioactive compounds and their health benefits: A review. J. Food Process. Technol..

[29] Roy A.J., Prince P.S.M. (2013). Preventive effects of p-coumaric acid on cardiac hypertrophy and alterations in electrocardiogram, lipids, and lipoproteins in experimentally induced myocardial infarcted rats. Food Chem. Toxicol..

[30] Lee S.J., Mun G.I., An S.M., Boo Y.C. (2009). Evidence for the association of peroxidases with the antioxidant effect of p-coumaric acid in endothelial cells exposed to high glucose plus arachidonic acid. BMB Rep..

[31] Seok J.K., Kwak J.Y., Seo H.H., Suh H.J., Boo Y.C. (2015). Effects of *Bambusae caulis* in Taeniam extract on the UVB-induced cell death, oxidative stress and matrix metalloproteinase 1 expression in keratinocytes. J. Soc. Cosmet. Sci. Korea.

[32] Peng J., Zheng T.-T., Liang Y., Duan L.-F., Zhang Y.-D., Wang L.-J., He G.-M., Xiao H.-T. (2018). p-Coumaric acid protects human lens epithelial cells against oxidative stress-induced apoptosis by MAPK signaling. Oxidative Med. Cell. Longev..

[33] Guglielmi F., Luceri C., Giovannelli L., Dolara P., Lodovici M. (2003). Effect of 4-coumaric and 3,4-dihydroxybenzoic acid on oxidative DNA damage in rat colonic mucosa. Br. J. Nutr..

[34] Garrait G., Jarrige J.F., Blanquet S., Beyssac E., Cardot J.M., Alric M. (2006). Gastrointestinal absorption and urinary excretion of trans-cinnamic and p-coumaric acids in rats. J. Agric. Food Chem..

[35] Žilić S., Šukalović V.H.-T., Dodig D., Maksimović V., Maksimović M., Basić Z. (2011). Antioxidant activity of small grain cereals caused by phenolics and lipid soluble antioxidants. J. Cereal Sci..

[36] Shen Y., Song X., Li L., Sun J., Jaiswal Y., Huang J., Guan Y. (2019). Protective effects of p-coumaric acid against oxidant and hy-perlipidemia-an in vitro and in vivo evaluation. Biomed. Pharmacother..

[37] Sakamula R., Thong-asa W. (2018). Neuroprotective effect of p-coumaric acid in mice with cerebral ischemia reperfusion injuries. Metab. Brain Dis..

[38] Akdemir F.N.E., Albayrak M., Çalik M., Bayir Y., Gülçin I. (2017). The protective effects of p-coumaric acid on acute liver and kidney damages induced by cisplatin. Biomedicines.

[39] Zhang L.-J., Huang H.-T., Huang S.-Y., Lin Z.-H., Shen C.-C., Tsai W.-J., Kuo Y.-H. (2015). Antioxidant and anti-inflammatory phenolic glycosides from *Clematis tashiroi*. J. Nat. Prod..

[40] Pragasam S.J., Venkatesan V., Rasool M. (2013). Immunomodulatory and anti-inflammatory effect of p-coumaric acid, a common dietary polyphenol on experimental inflammation in rats. Inflammation.

[41] Zhao Y., Liu J., Liu C., Zeng X., Zhao X.L.A.J. (2016). Anti-inflammatory effects of p-coumaric acid in LPS-stimulated RAW264.7 cells: Involvement of NF-κB and MAPKs pathways. J. Med. Chem..

[42] Sabitha R., Nishi K., Gunasekaran V.P., Annamalai G., Agilan B., Ganeshan M. (2019). p-Coumaric acid ameliorates ethanol–induced kidney injury by inhibiting inflammatory cytokine production and NF–κB signaling in rats. Asian Pac. J. Trop. Med..

[43] Urfalioğlu A., Yazar F.M., Bilal B., Tolun F.İ., Öksüz H., Boran Ö.F., Gözen Ö. (2017). The effect of p-coumaric acid and ellagic acid on the liver and lungs in a rat model of sepsis. Asian Biomed..

[44] Janicke B., Onning G., Oredsson S.M. (2005). Differential effects of ferulic acid and p-coumaric acid on S phase distribution and length of S phase in the human colonic cell line Caco-2. J. Agric. Food Chem..

[45] Bouzaiene N.N., Jaziri S.K., Kovacic H., Chekir-Ghedira L., Ghedira K., Luis J. (2015). The effects of caffeic, coumaric and ferulic acids on proliferation, superoxide production, adhesion and migration of human tumor cells in vitro. Eur. J. Pharmacol..

[46] Shailasree S., Venkataramana M., Niranjana S.R., Prakash H.S. (2015). Cytotoxic effect of p-coumaric acid on inducing apoptosis and autophagy. Mol. Neurobiol..

[47] Min S.J., Lim J.Y., Kim H.R., Kim S.J., Kim Y. (2015). Sasaquel paertensis leaf extract inhibits colon cancer by regulating cancer cell stemness in vitro and in vivo. Int. J. Mol. Sci..

[48] Radwan M.M., Badawy A., Zayed R., Hassanin H., El Sohly M.A., Ahmed S.A. (2015). Cytotoxic flavone glycosides from Sola-num elaeagnifolium. Med. Chem. Res..

[49] Kong C.-S., Jeong C.-H., Choi J.-S., Kim K.-J., Jeong J.-W. (2012). Antiangiogenic effects of p-coumaric acid in human endothelial cells. Phytother. Res..

[50] Amalan V., Vijayakumar N., Ramakrishnan A. (2015). p-Coumaric acid regulates blood glucose and antioxidant levels in strep-tozotocin induced diabetic rats. J. Chem. Pharm. Res..

[51] Amalan V., Vijayakumar N., Indumathi D., Ramakrishnan A. (2016). Antidiabetic and antihyperlipidemic activity of p-coumaric acid in diabetic rats, role of pancreatic GLUT 2: In vivo approach. Biomed. Pharmacother..

[52] Zabad O.M., Samra Y.A., Eissa L.A. (2019). p-Coumaric acid alleviates experimental diabetic nephropathy through modulation of toll like receptor-4 in rats. Life Sci..

[53] Adisakwattana S., Sookkongwaree K., Roengsumran S., Petsom A., Ngamrojnavanich N., Chavasiri W., Deesamer S., Yibchok-Anun S. (2004). Structure–activity relationships of trans-cinnamic acid derivatives on α-glucosidase inhibition. Bioorg. Med. Chem. Lett..

[54] Yoon S.-A., Kang S.-I., Shin H.-S., Kang S.-W., Kim J.-H., Ko H.-C., Kim S.-J. (2013). p-Coumaric acid modulates glucose and lipid metabolism via AMP-activated protein kinase in L6 skeletal muscle cells. Biochem. Biophys. Res. Commun..

[55] Lima L.C., Buss G.D., Ishii-Iwamoto E.L., Salgueiro-Pagadigorria C., Comar J.F., Bracht A., Constantin J. (2006). Metabolic effects of p-coumaric acid in the perfused rat liver. J. Biochem. Mol. Toxic..

[56] An S.M., Lee S.I., Choi S.W., Moon S.W., Boo Y.C. (2008). p-Coumaric acid, a constituent of Sasa quelpaertensis Nakai, inhibits cellular melanogenesis stimulated by α-melanocyte stimulating hormone. Br. J. Dermatol..

[57] An S.M., Koh J.-S., Boo Y.C. (2010). p -Coumaric acid not only inhibits human tyrosinase activity in vitro but also melanogenesis in cells exposed to UVB. Phytother. Res..

[58] Boo Y.C. (2019). p-Coumaric acid as an active ingredient in cosmetics: A review focusing on its antimelanogenic effects. Antioxidants.

[59] Lim J.-Y., Ishiguro K., Kubo I. (1999). Tyrosinase inhibitory p-coumaric acid from ginseng leaves. Phytother. Res..

[60] An S.M., Lee S.J., Koh J.S., Park K., Boo Y.C. (2010). Effects of plant extract-containing creams on UV radiation-induced inflammatory responses in mice. J. Soc. Cosmet. Sci. Korea.

[61] Song K., An S.M., Kim M., Koh J.-S., Boo Y.C. (2011). Comparison of the antimelanogenic effects of p-coumaric acid and its methyl ester and their skin permeabilities. J. Dermatol. Sci..

[62] Reagan-Shaw S., Nihal M., Ahmad N. (2008). Dose translation from animal to human studies revisited. FASEB J..

[63] Kheiry M., Dianat M., Badavi M., Mard S.A., Bayati V. (2020). Does p-coumaric acid improve cardiac injury following LPS-induced lung inflammation through miRNA-146a activity?. Avicenna J. Phytomed..

[64] Zhu H., Liang Q.-H., Xiong X.-G., Wang Y., Zhang Z.-H., Sun M.-J., Lu X., Wu D. (2018). Anti-inflammatory effects of p-coumaric acid, a natural compound of Oldenlandiadiffusa, on arthritis model rats. Evid. Based Complement. Altern. Med..

[65] Jaganathan S.K., Supriyanto E., Mandal M. (2013). Events associated with apoptotic effect of p-coumaric acid in HCT-15 colon cancer cells. World J. Gastroenterol..

[66] Kumar S., Pandey A.K. (2015). Free radicals: Health implications and their mitigation by herbals. Br. J. Med. Med. Res..

[67] Pizzino G., Irrera N., Cucinotta M., Pallio G., Mannino F., Arcoraci V., Squadrito F., Altavilla D., Bitto A. (2017). Oxidative stress: Harms and benefits for human health. Oxid. Med. Cell. Longev..

[68] Dröge W. (2002). Free radicals in the physiological control of cell function. Physiol. Rev..

[69] Halliwell B., Gutteridge J.M. (2015). Free Radicals in Biology and Medicine.

[70] Hansen J.M., Go Y.-M., Jones D.P. (2006). Nuclear and mitochondrial compartmentation of oxidative stress and redox signaling. Annu. Rev. Pharmacol. Toxicol..

[71] Chen L., Deng H., Cui H., Fang J., Zuo Z., Deng J., Li Y., Wang X., Zhao L. (2018). Inflammatory responses and inflammation-associated diseases in organs. Oncotarget.

[72] Medzhitov R. (2010). Inflammation 2010: New adventures of an old flame. Cell.

[73] Sikora E., Scapagnini G., Barbagallo M. (2010). Curcumin, inflammation, ageing and age-related diseases. Immun. Ageing.

[74] Sesti G. (2006). Phathophysiology of insulin resistance. Best Pract. Res. Clin. Endocrinol. Metab..

[75] Bhattarai G., Min C.-K., Jeon Y., Bashyal R., Poudel S.B., Kook S., Lee J. (2019). Oral supplementation with p -coumaric acid protects mice against diabetes-associated spontaneous destruction of periodontal tissue. J. Periodontal Res..

[76] Abdel-Moneim A., Yousef A.I., El-Twab S.M.A., Reheim E.S.A., Ashour M.B. (2017). Gallic acid and p-coumaric acid attenuate type 2 diabetes-induced neurodegeneration in rats. Metab. Brain Dis..

[77] Leto D., Saltiel A. (2012). Regulation of glucose transport by insulin: Traffic control of GLUT4. Nat. Rev. Mol. Cell Biol..

[78] Brenner M., Hearing V.J. (2008). The protective role of melanin against UV damage in human skin. Photochem. Photobiol..

[79] Slominski A., Tobin D., Shibahara S., Wortsman J. (2004). Melanin pigmentation in mammalian skin and its hormonal regulation. Physiol. Rev..

[80] Gilchrest B.A., Eller M.S. (1999). DNA photodamage stimulates melanogenesis and other photoprotective responses. J. Investig. Dermatol. Symp. Proc..

[81] Garcia-Borron J.C., Sanchez M.C.O., Borovansky J., Riley P.A. (2011). Biosynthesis of melanin. Melanins and Melanosomes: Biosynthesis, Structure, Physiological and Pathological Functions.

[82] De Smet P.A.G.M. (1997). The role of plant-derived drugs and herbal medicines in healthcare. Drugs.

[83] Boz H. (2015). p-Coumaric acid in cereals: Presence, antioxidant and antimicrobial effects. Int. J. Food Sci. Technol..

